# Maternal Healthcare Providers in Uttar Pradesh, India: How to Position Informal Practitioners within the System?

**Published:** 2014-12

**Authors:** Chesta Sharma, Kanchan Mukherjee

**Affiliations:** School of Health Systems Studies, Tata Institute of Social Sciences, Mumbai, India

**Keywords:** Maternal Healthcare, Informal Providers, Health Human Resources, India

## Abstract

**Objective:** To understand the knowledge and services of informal providers and to explore their role in addressing the human resource gap in Uttar Pradesh, India, within the context of maternal health.

**Materials and methods:** The study is exploratory in nature, conducted in four blocks of four districts of Uttar Pradesh state, India. Semi-structured interviews were conducted with 114 informal providers.

**Results:** More than one-third (38%) providers have some formal education and unrecognized degrees. Approximately three-fourths (74%) of them have more than 5 years of work experience. They also provide delivery and in-patient services and have basic equipment available. However, they lack essential knowledge about maternal health. They have mixed opinion about their contribution towards maternal health but the only ones available. Therefore, despite lacking requisite knowledge, training and services, they become indispensable due to lack of emergency and timely public health services, and being the only ones existing in the community.

**Conclusion:** Informal sector practitioners are a critical link in reaching out to population for health services in developing countries. As opposed to the general notion, they possess years of formal education, experience, informal trainings along with trust of communities. Thus, it becomes important to accept their presence and manage them to the best of their abilities even for specialized care like maternal health.

## Introduction

India has a complex healthcare system due to mixed ownership patterns, different types of providers and different systems of medicine. Unfortunately, the distribution of healthcare is uneven, where bulk of the service is available in urban areas and is dominated by the private sector.

India has about 1.4 million trained medical practitioners, out of which 0.7 million are graduate allopath. However, about 74% of these doctors live in urban areas, thereby serving only 28% of the population (1). The global shortage of health workers approached 4.3 million in 2006, the greatest being in South-East Asia, dominated by Bangladesh, India and Indonesia (2). Indian healthcare system has an intensive institutional network and presence of a diverse human resource. However, the public health system suffers from shortages, imbalances, mal-distribution, poor work environments, low personnel productivity, numerous vacant posts, high staff turnover, loss of personnel to private sector, and migration of workers to urban areas or overseas (3).

Indian policies and programs constantly revisit and revise the focus areas to improve the state of maternal and child health; though the importance and role of health service providers is largely neglected. Most strategies focus on building cadres or waiting for educational institutions to yield trained human resources. 

Rhode & Viswanathan cited in Gidwani suggests that there are approximately 1.25 million unqualified rural medical practitioners in India (4). The public healthcare system in India is loaded with problems; hence the rural private practitioner is by default the primary care provider and the first point of contact for the poor.

A study done in northern Karnataka (India) clearly states rural medical practitioner (RMP) as a critical resource for care, referral and advice. They have linked networks with formal providers both in the public and private sector. They are seen with limited expertise while having social relationships with communities (5). A review by Shah et al. quoted by Bloom et al. identified 70 studies that talk in favor of informal providers being part of impactful interventions (6).

Uttar Pradesh is one of the most populous states in India with a population of 199 million and one of the highest maternal mortality ratio (MMR), i.e. 359, about 1.7 times the national average (7, 8). In Uttar Pradesh, about 78% deliveries happen at home, less than one‐third receive ante‐natal care and less than 15% receive post‐natal care through the public health system (9). Hence, it is essential to understand the kind of providers that are being accessed for maternal health. It is also important to understand the providers’ perspective of their own position/role in addressing the issue of health human workforce.

This study attempts at understanding the knowledge and services of informal providers and exploring their role in addressing the maternal health human resource gap across continuum of care in Uttar Pradesh, India.

## Materials and methods

The study is exploratory in nature and was conducted during March to May 2013. It was conducted in 16 villages in four blocks of four districts of Uttar Pradesh, India.

A list of informal providers was generated from focus group discussions with women, husbands and mother-in-laws. The discussions focused on continuum of care in maternal health and explored various healthcare providers that were accessed for checkups, complications, deliveries and other requirements. Semi-structured interviews were conducted with the providers. Informed consent was obtained from all participants.

A total of 114 providers were interviewed. Of the 114 providers, 81 were based in villages, 24 in semi-rural areas, four in semi-urban areas, and five in urban areas.

The study adopts the working definition of an informal healthcare provider as an individual who has no recognized healthcare training or has training that has been recognized in the past, but is not being recognized in the present. He/she is being paid in kind/cash. He/she is not registered with any formal organization, and hence is not being regulated by one (10). This category also includes formal public healthcare providers who cross-practice in the private sector and provide services that they are not trained or qualified for.

## Results

Approximately 38% providers were educated up to 10^th ^standard or higher, and some even possessed unrecognized professional degrees (Table 1). 

**Table 1:** Educational and professional qualifications of providers

Education			Professionals^d^			Cross practice^f^		
**Illiterate**	**16**	**14%**	**None**	**91**	**79.8%**	**None**	**106**	**93%**
**8** ^th^ **Std.**	**01**	**0.9%**	**TBA** e	**10**	**8.8%**	**ANM**	**04**	**3.5%**
**10** ^th^ ** Std.**	**43**	**37.7%**	**Electrohomeopath**	**05**	**4.4%**	**Staff nurse**	**01**	**0.9%**
**12** ^th^ **Std.**	**28**	**24.6%**	**AyurvedRatan**	**04**	**3.5%**	**Health inspector**	**01**	**0.9%**
**B.A.** a	**18**	**15.8%**	**Dai**	**03**	**2.6%**	**Lab technician**	**01**	**0.9%**
**B.Sc.** b	**05**	**4.4%**	**Faith healer**	**01**	**0.9%**	**ASHA** g	**01**	**0.9%**
**M.A.** c	**03**	**2.6%**						

*All percentages are approximates

a Bachelor of arts;

b Bachelor of science;

c Master of arts;

d Informal providers with unrecognized degrees/ degrees that are no more recognized;

e Trained birth attendants;

f Cross practice include public health workers who are providing services in the private sector and may or may not be qualified to provide those services;

g Accredited social health activist;

Approximately 44% providers worked only at their current place; remaining had worked at other places as well. Approximately 26% providers had five years or less work experience, but majority had six years or more, thereby increasing their rapport and experience with the community (Table 2).

**Table 2:** Work experience at the current and other places

**Work** ** experience at current place**			
5 years or less	30	26.3%	
6-10 years	19	16.7%	
11-15 years	35	30.7%	
16-20 years	11	9.7%	
21-35 years	11	9.7%	
Whole Life	08	7%	
**Work experience at other place**			
None	50		43.9%
5 years or less	43		37.7%
6-15 years	19		16.7%
21-25 years	02		1.8%

Approximately 41% providers are giving 24-hour services as they live in the same villages. A small (19.3%) but sizeable proportion is also providing delivery services (Table 3). Pharmacy is available with 47.4% providers. Basic equipment like auto disposable syringes (57%), sphygmomanometer (66.7%), stethoscope (70.2%) and thermometer (67.5%) are also available with them. Few are engaged in providing pregnancy tests (8.8%) and treatment for complications (14.9%) as well (Table 4).

***Knowledge Levels of Informal Providers***

Providers were assessed for their knowledge on maternal health to understand their capabilities to contribute towards the improvement of maternal health. Only 29% providers could identify third trimester to one week post-delivery phase being the most likely phase for maternal death to occur (11).

**Table 3:** Number of service hours, delivery services, delivery services in the night, in-patient facilities, laboratory services, pharmacy, and ultrasound facilities (out of 114)

**Services **		
24-hour services	47	41.2%
In-patient facility	05	4.4%
Laboratory services	03	2.6%
Pharmacy	54	47.4%
Ultrasound	00	00
Delivery services	22	19.3%
Delivery services in night	20	17.5%
Complicated deliveries (out of 22)	19	16.7%
C-sections (out of 22)	00	00

Anemia, malnutrition, lack of care, poor diet, lack of food, bleeding and weakness, and high blood pressure were listed as the commonest causes of maternal death (Table 5). Vomiting, anemia and lack of appetite were listed as commonest antenatal complications; bleeding, delayed labor and pain as commonest intra-natal complications; and hemorrhage, infection and anemia as commonest post-natal complications.It is important to note that 36% providers did not know of any antenatal complications, 45% did not know of any intra-natal complications and 41% did not know of any post-natal complications.

The providers were asked to rank hemorrhage, infection, unsafe abortion, eclampsia and obstructed labor as most to least common cause of maternal death. Hemorrhage was correctly identified by 32.5% providers as the topmost cause of maternal death followed by infection (19.3%) as the second most common cause of maternal death. However, few could identify obstructed labor (7.9%) as third most common cause, eclampsia (1.8%) as fourth most common cause, and unsafe abortions (7%) as fifth most common cause of maternal death (Table 4) (12).

**Table 4:** Availability of equipment and services

**Equipment available (out of 114)**				**Services available (out of 114)**			
1	Auto-disposable syringes	65	57%	1	Hemoglobin tests	03	2.6%
2	Sphygmomanometer	76	66.7%	2	Pregnancy tests	10	8.8%
3	Stethoscope	80	70.2%	3	Iron and folic acid tablets	32	28.1%
4	Weighing machine	32	28.1%	4	Tetanus injections	33	29%
5	Hemoglobin-meter	05	4.4%	5	Counseling on diet and rest	38	33.3%
6	Vaccines	33	29%	6	Dietary supplements	13	11.4%
7	Thermometer	77	67.5%	7	ANC complications treatment	17	14.9%
				8	PNC complications treatment	17	14.9%

**Table 5:** Ranking causes of maternal death

**Ranking causes of maternal death (each out of 114)**		
Hemorrhage as the most common cause	37	32.5%
Infection as the second most common cause	22	19.3%
Obstructed labor as the third most common cause	09	7.9%
Eclampsia as the fourth most common cause	02	1.8%
Unsafe abortions as the fifth most common cause	08	7%

***Role of providers in improving maternal health***

Some feel that government workers and facilities are improving and people are becoming aware to access them. One said, “*Pregnant women know a lot of things now. ANMs (Auxiliary Nurse Midwives) and ASHAs (Accredited Social Health Activist) are also working nicely. They know a lot and make women understand everything.*” They also emphasized on improving the government facilities to improve maternal health. Another said, “*If government facilities are increased, then nobody would need people like us.*” 

However, some feel that government workers are corrupt, have private practices and services are incomplete. One said, “*In the hospitals, they work according to their own wish. People are busy making money. Everybody has private clinics. CHCs (Community Health Center) ANM take 500 rupees, while another ANM takes 3000 rupees for a complicated delivery and 1000 rupees for a normal delivery.*” Another said, “*Government facilities are not complete. All equipmentis not there. Their behavior should improve too.*”

Some feel that government programs are good, but not managed properly. They believe that linking them with private health system might work better. One said, “*If the government programs are managed properly they are all very good. Due to lack of facilities, they remain only on paper. The workers have shortcomings. Government should link its program with private doctors; it will benefit the public a lot.*”

***Role of Dais (Traditional Birth Attendants)***

Some feel that dais are essential and do good work, while others feel they are harmful. One said, “*Dais work from their heart. They have wonderful experience.*” Another said, “*There should be professional nurses in hospitals. Dais work in unhygienic conditions. They press the abdomen and harm the placenta. They do not know about any complications.*” Some feel that they should be trained. One said, “*Dai training should happen. It should be done on experience basis.*” 

***Role of Quacks***

Many providers know that they are harmful to patients and should be banned. One said, “*At times patients suffer loss.*” Another said, “*Ideally they should be banned.*” However, they also feel that it is difficult to ban them, as they are the only ones available in villages. One said, “*Many times, the government hospital is closed. If they come to us, at least we give them medicines even if we take money.*”

Some feel that though government programs are good, they are not reaching everywhere. Therefore, government should have specific criteria to select better and experienced informal providers and train them to provide services in the interiors and for referral. As part of such training, they may be allowed to assist trained doctors. One of them said, “*Qualified doctors have in-depth knowledge. Government facilities are not giving emergency services; government schemes are not reaching people. They should analyze people who are practicing and then make a selection amongst them.*” Another said, “*Everybody should be given 20 villages, let us see them, take a daily report, this is the only system that can be successful in interiors. Government should run it like this in interiors. Mobile units should be installed.*” Another said, “*They should receive knowledge and training. Nobody comes here to treat. Some power should be given, so that they can assist trained doctors and work with them. They can treat patients in the village.*”

However, some feel that it is an impossible task to link informal providers with the public health system and if done, it won’t be beneficial. One said, “*It is not possible, there are no standards, there are no qualifications.*” Another said, “*If training is given to them, they would run after money. It is not possible. Government will not do it. How can an MBBS and a non-MBBS be brought together?*” 

Informal providers are very critical in their perceived role to improve maternal health status. Majority do not accept themselves having an exclusive role, but express interest towards engaging with the system. They acknowledge that the current health programs and workforce are good, if managed properly. They feel that system would progress if improvements are made and shortcomings are addressed. Few see it as a possible solution to better maternal health status in the country. They do reflect on their role, which varies from beneficial to harmful. Due to lack of knowledge and training, they accept their services and practices as limited and thus could be harmful to people. But, they also know due to lack of emergency and timely public health services, they are the only ones existing in the community making them indispensable. Thus, some suggest trainings based on a selection criterion for their own benefit and also for the benefit of the community.

## Discussion

Majority of the doctors who practice in rural areas are unlicensed or informally trained and are usually the first point of contact. The Bhore Committee dissenters argued that it would be impossible to get rid of the licentiate and indigenous practitioners who are extensively present in today’s rural India (12). These providers serve their neighbouring areas and provide easy and accessible healthcare in areas with significant transportation problems.

Most villages do not have government health facilities. Approximately 74% providers in the study have six or more years of experience within their own community and approximately 56% even have experience outside their community. Approximately 41% providers give 24 hours service as they live in the same villages. Despite not being trained and not having enough knowledge about maternal health problems, they provide treatment across continuum of care. Most of them have their one-room setups with basic equipment and services. Many have practiced under formal health providers and follow a referral chain with them, wherein they refer difficult or complicated cases to known higher centers.

It is important to understand the gap in knowledge and training of providers who are giving maternal health services within the community. This study clearly demonstrates informal sector practitioners as one of the essential providers of maternal healthcare but lacking requisite knowledge. It brings together the three important facts of informal providers as stated by Olson that they are respectable members of their own communities, have some formal education and unrecognized or informal training (13).

## Conclusion

India leads the world in total number of maternal deaths. The intra-regional disparities within the country are even higher, wherein a state like Uttar Pradesh has higher MMR than the national figure (8). The country is far from ideal provider-population ratios, but Uttar Pradesh falls back further. Many women in the state access public health facilities, but a high percentage of women access private health facilities as well. Therefore, it becomes critical to understand the contribution of the private sector, which is not only formal but informal as well. 

This study highlights that such practitioners are not just formally educated, but may hold graduate or postgraduate degrees along with some informal or unrecognized degrees and training. However, they lack requisite knowledge to provide maternal health services, but sometimes make up with experience (14).

The cultural and social construct of communities, especially in a country like India, makes them access dais, Trained Birth Attendants (TBAs) and informal sector practitioners who are readily available for maternal health services. Therefore, it is important to recognize their presence in states like Uttar Pradesh where the birth rate and MMR are high and public health system is not at par with the national standards. It is also important to understand their utilization to the best of their capabilities not only for basic health services but also for specialized cases like maternal health as they have extensive experience, informal training, some formal education and most importantly trust of communities.
